# Effects of 405-, 532-, 650-, and 940-nm wavelengths of low-level laser therapies on orthodontic tooth movement in rats

**DOI:** 10.1186/s40510-020-00343-3

**Published:** 2020-12-01

**Authors:** Hasibe Baser Keklikci, Ahmet Yagci, Arzu Hanim Yay, Ozge Goktepe

**Affiliations:** 1 Eskisehir Oral and Dental Health Hospital, Yenikent, Piri Reis St. No. 28, Odunpazarı, 26050 Eskisehir, Turkey; 2 Department of Orthodontics, Faculty of Dentistry, Erciyes University, Kayseri, Turkey; 3 Department of Histology and Embryology, Faculty of Medicine, Erciyes University, Kayseri, Turkey; 4 Genome and Stem Cell Center (GENKOK), Erciyes University, Kayseri, Turkey

**Keywords:** Low-level laser therapy (LLLT), Accelerated orthodontic tooth movement, TRAP, ALP

## Abstract

**Background:**

Investigating the effects of 405-nm, 532-nm, 650-nm, and 950-nm wavelengths of LLLTs (low-level laser therapies) on the orthodontic tooth movement in rats by using histological and immunohistochemical methods. Forty-five Wistar albino rats were randomly divided into 5 groups: control group (positive control: the left maxillary 1st molar side; negative control: the right maxillary 1st molar side), 405 nm LLLT group (Realpoo), 532 nm LLLT group (Realpoo), 650 nm LLLT group (Realpoo), and 940 nm LLLT group (Biolase). The left maxillary 1st molar teeth of all rats were applied mesially 50-g force. Starting from the 1st day, 48 h intervals, LLLT was applied in continuous wave mode and in contact with the tissue. The application area was approximately 1 cm^2^. The lasers were performed for 3 min on each surface (buccal, palatal, mesial), totally 9 min (total dose 54 J/cm^2^). The amount of the molar mesialization, the bone area between the roots, PDL (periodontal ligament) measurements, TRAP (tartrate-resistant acid phosphatase), and ALP (alkaline phosphatase) immunoreactivity intensity were calculated.

**Results:**

The amount of the molar mesialization was significantly higher in the 650 nm LLLT group (mean 0.878 ± 0.201 mm; 95% CI (confidence interval) 0.724 and 1.032) than in the groups of positive control (mean 0.467 ± 0.357 mm; 95% CI 0.192 and 0.741) and 405 nm LLLT (mean 0.644 ± 0.261 mm; 95% CI 0.443 and 0.845) (*p* < 0.001). There were significant differences in the PDL-mesial (*p* = 0.042) and PDL-distal (*p* = 0.007) regions between the groups. The immunoreactivity intensity for TRAP-mesial was significantly higher in the positive control group (mean 109,420.33 ± 8769.17; 95% CI 100,217.65 and 118,623.02) than in the 405 nm (mean 91,678.83 ± 7313.39; 95% CI 84,003.9 and 99,353.77) and the 650 nm LLLT (mean 87,169.17 ± 4934.65; 95% CI 81,990.56 and 92,347.77) groups (*p* = 0.002). There was no statistically significant difference between the groups on immunoreactivity intensity with ALP staining.

**Conclusions:**

The results of this study show that LLLT with 650-nm wavelength increases orthodontic tooth movement more than 405-nm, 532-nm, and 940-nm LLLTs. The 940-nm and 650-nm LLLTs also increase the bone area between the roots by more than 405-nm and 532-nm wavelengths.

## Background

For the patients who have an indication for orthodontic treatment, the biggest concerns are the long duration of the therapy and the pain they will suffer during this process [1]. The prolongation of the orthodontic treatment period may lead to many undesirable conditions such as reduced patient cooperation, caries formation, periodontal diseases, and root resorption. Therefore, researchers are working on methods that will accelerate orthodontic tooth movement and shorten the duration of treatment.

In the literature, the methods that accelerate tooth movement are under four main headings as traditional orthodontic biomechanical methods, surgical, chemical, and physical applications [2]. Because the effect of traditional orthodontic methods on accelerating tooth movement is limited, surgical-assisted methods require an invasive procedure and may be painful, and chemical methods show systemic side effects other than their local effects; clinicians tend to be more interested in physical methods [3, 4]. Because it is easy to apply and requires only a few equipments, LLLT (low-level laser therapy) is one of the most popular techniques among them.

LLLT is considered to be effective in controlling pain, modulating inflammation, accelerating the growth of new tissue, and increasing wound healing. When light enters the tissue and is absorbed, biochemical processes are triggered that lead to the activation of the mitochondrial chain and then mainly to the increase in the production of ATP (adenosine triphosphate), NO (nitric oxide), and a small amount of ROS (reactive oxygen species). As a result, LLLT accelerates the activities on a cell and affects the process on the tissue level [5]. The laser-induced analgesia is thought to operate on a variety of local and systemic pathways including the inhibition of axonal depolarization; selective reduction of acute inflammatory mediators such as prostaglandins, IL1-β (Interleukin 1 beta), IL-6 (Interleukin 6), and TNF-α (tumor necrosis factor alpha); vasodilatation; and improved lymphatic drainage [6].

Receptor activator of nuclear factor kappa-B ligand (RANKL) and its receptor RANK (receptor activator of nuclear factor kappa-B) present a regulatory function in bone homoeostasis [7]. The available limited evidence suggests that LLLT increases the expression of both RANK and RANKL [8] and may have a role in accelerating orthodontic tooth movement [9–11].

Besides the studies showing that LLLT accelerates the movement of the teeth [12, 13], there are also the ones that conclude it slows down the shift [14] or the effect of LLLT is insignificant [15]. In many studies, different doses or different application times of the same wavelength laser were investigated. In few ones, two different wavelengths were applied in the same particular research, but many parameters such as application mode, power density, energy density, frequency of application, and experimental period of lasers vary between the experimental groups. Therefore, the interpretation of these results is a complex work, and it is not possible to compare the statistics of these studies with each other in a healthy analytic manner.

To accelerate tooth movement and shorten the duration of orthodontic treatment, it is very important to understand tooth movement histologically and immunohistochemically. In orthodontic tooth movement, a high rate of TRAP (tartrate-resistant acid phosphatase) concentration is observed in destructive cells in bone and root resorption areas [16]. TRAP is an enzyme secreted from osteoclasts during active bone destruction. Therefore, TRAP staining is used to identify resorptive cells that cannot be identified by routine histological staining in orthodontic tooth movement and orthodontic root resorption [17]. ALP (alkaline phosphatase) indicates osteoblastic activity during bone formation. ALP is produced at very high concentrations during the construction phase of the bone cycle and provides a good idea of the overall bone-building activity.

## Methods

The aim of this study is to investigate the effects of low-level lasers of the 405-nm, 532-nm, 650-nm, and 940-nm wavelengths, on the tooth movement rate by using histological and immunohistochemical methods.

The number of animals to be used was determined as 90% power and *p* = 0.05 impacts according to power analysis, and a total of 45 subjects in each group 9 subjects. Forty-five Wistar albino rats (8 weeks old; weight, 140.98 ± 12.86 g) were in polycarbonate cages at an average temperature of 22 ± 2 °C, 12 h light and 12 h dark. They were fed ad libitum with a standard laboratory diet and tap water. No food restriction was made.

### Groups

**Fig. 1:**
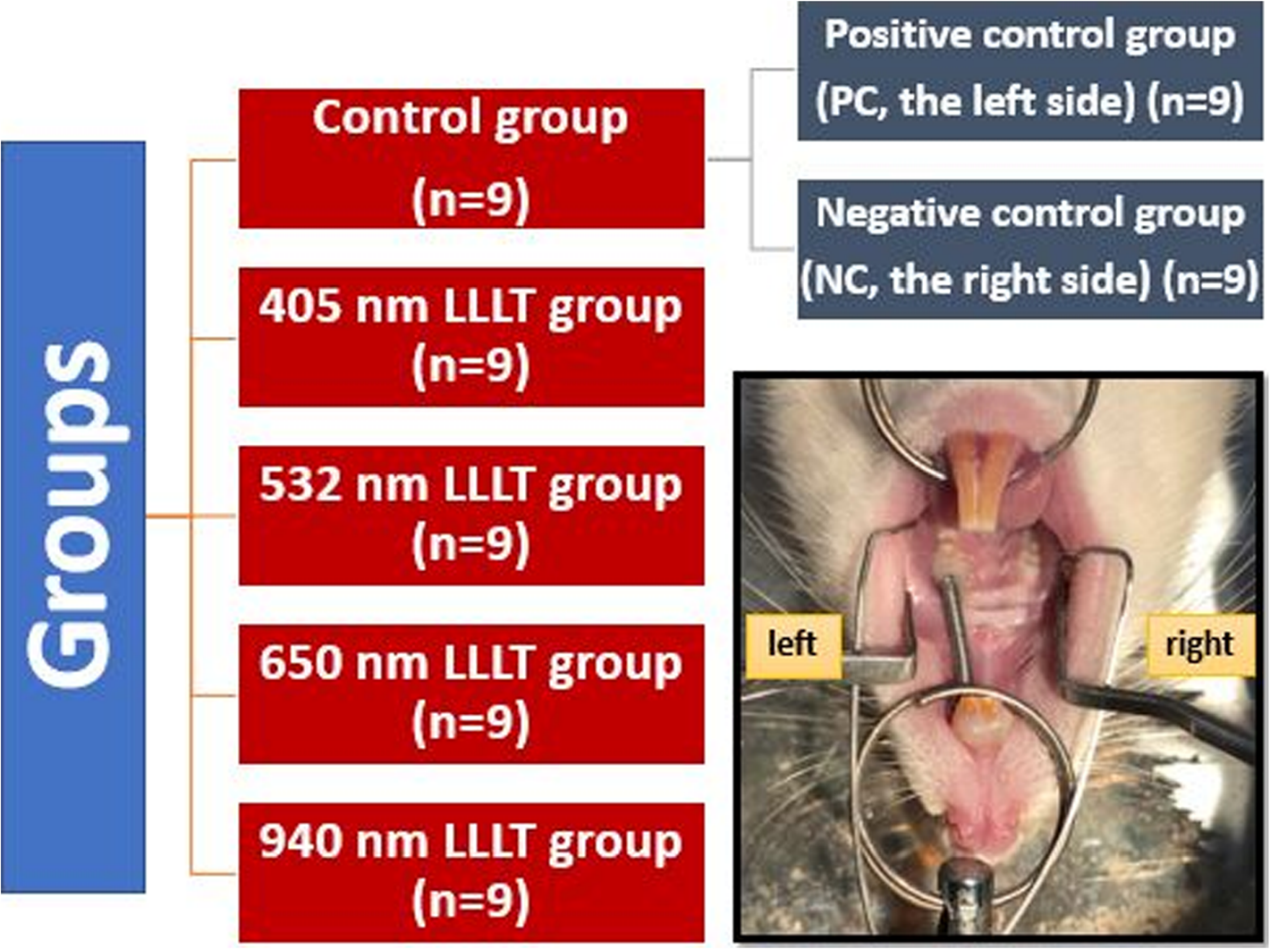
Schematic representation of the groups. The image of nickel-titanium was applied to the incisor and 1st molar teeth with 50-g force

Rats were divided into five study groups randomly: control group, 405 nm LLLT group, 532 nm LLLT group, 650 nm LLLT group, and 940 nm LLLT group. In the control group, only experimental tooth movement was created, and no laser application was performed. In the control group, the left maxillary 1st molars were used as a positive control (PC) group and the right maxillary 1st molars were used as a negative control (NC) group (Fig. 1).

### Experimental tooth movement

To obtain experimental tooth movement, nickel-titanium closed-coiled springs were placed between the maxillary incisor teeth and the left maxillary 1st molar teeth of the rats to apply 50-g force. The experiment period was 14 days. No activation was made for the tooth movement during the experimental period; only the robustness of the apparatus was checked (Fig. 1).

### Laser irradiation

**Fig. 2:**
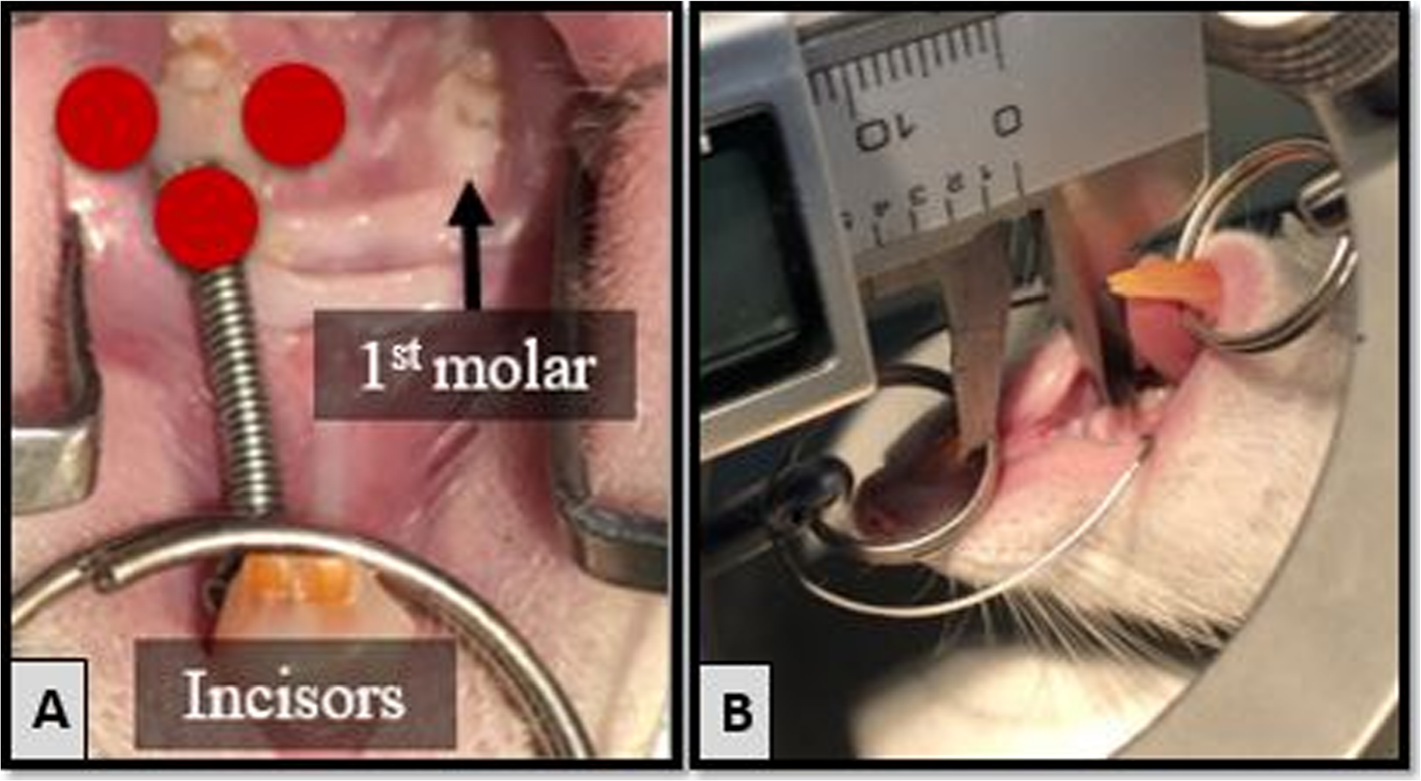
**a** Schematic representation of low-level laser-treated areas with red circles (buccal, palatal, and mesial). **b** Measurement of the distance between the incisor and the 1st molar teeth with a digital caliper

Diode laser modules (405 nm (Realpoo, RP100AD405-10BD, Jilin, China); 532 nm (Realpoo, M532D100, Jilin, China); 650 nm (Realpoo, RP100AD650-10 BC, Jilin, China), and 940 nm (Biolase, Epic 10^TM^, Ingbert, Germany)) with 100 mW/cm^2^ output power calibrated with the calibration device (Apogee Solar Radiation MP-200). Starting from the 1st day, with 48 h intervals, LLLT was applied in a continuous wave mode and in contact with the tissue. The application area was approximately 1 cm^2^. The lasers were performed for 3 min on each surface (buccal, palatal, mesial), totally 9 min (total dose 54 J/cm^2^) (Fig. 2a).

### Anesthesia

All operations were carried out under general anesthesia, with an intraperitoneal injection of ketamine HCl (1.0 mg/kg; Alfamine®, Egevet, Turkey) and xylazine HCl (0.5 mg/kg; Rompun®, Bayer, Leverkusen, Germany) combination. For the purpose of checking the apparatus and equalizing the stress levels of general anesthesia on animals, the control group animals were also anesthetized with 48 h intervals, as in the laser groups.

### Measurement of the tooth movement

**Fig. 3:**
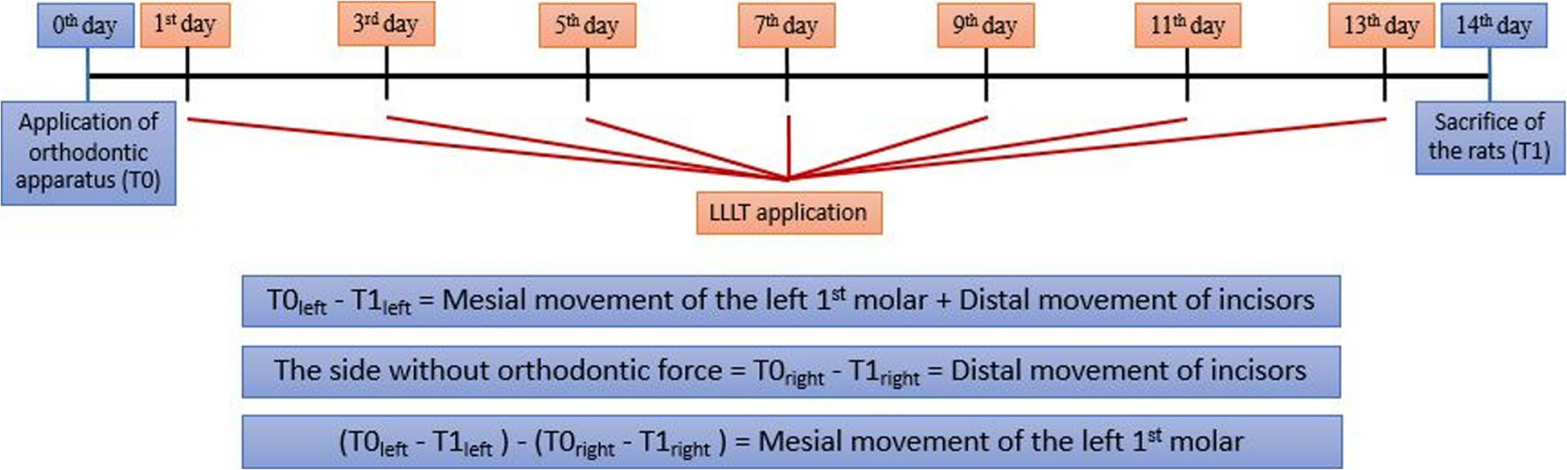
Demonstration of the study design and calculation of molar mesial movement

The distances between the incisor tooth (palatal side, gingival level) and the 1st molar tooth mesial surface (most convex point) in the maxilla were measured with a digital caliper with an accuracy of 0.01 mm on both the right and the left sides before the apparatus was applied (T0) and at the end of the experiment period (T1) (Fig. 2b). The measurements were repeated 3 times, and the average of these values was recorded. The difference between the left side initial length (T0_left_) and the length after tooth movement (T1_left_) was recorded. The same procedure was repeated for the right side. In order to determine the amount of molar mesialization, the difference in length on the right side (distal tipping amount of the incisors) was subtracted from the length difference on the left side (Fig. 3).

### Histological assessment

After the sacrification of the subjects, the tissues containing the tooth samples were put in a 10% formaldehyde solution before the examination. Tissues which were kept in formaldehyde for 72 h were decalcified in a decalcification solution containing acetic acid and embedded in paraffin. Serial sections of 5-μm-thick were stained with Masson’s trichrome for light microscopic histological examination. Images were analyzed by using a microscope (Olympus BX-51, Japan) for morphological observation of tissues.

For the histologic examinations, the mesial roots of the maxillary first molars were used. Images were obtained with × 10 magnification via the ImageJ software (http://imagej.nih.gov/ij/; provided in the public domain by the National Institutes of Health, Bethesda, MD, USA) and recorded. In the histological sections stained with Masson trichrome, the PDL distance between the root and the alveolar bone of the mesial root of the maxillary 1st molar tooth was measured from the cervical in the mesial (PDL-M; pressure side) and distal (PDL-D; tension side) regions of the root. In the same sections, the bone area between the mesial and mesiopalatinal roots of the maxillary 1st molar tooth was calculated by marking at 10X magnification with the ImageJ software program in the region that passes through the most apical points of the roots [18].

### Immunohistochemistry

In this study, the expression of TRAP and ALP in mesial root tissue of the maxillary 1st molar tooth was demonstrated immunohistochemically. Paraffin-embedded ovarian tissue sections of 5-μm-thick were dehydrated first in xylene and next in graded ethanol solutions. The slides were then blocked with 5% bovine serum albumin in phosphate-buffered saline (PBS) for 2 h. Then, IHC staining was performed by a standard avidin-biotin-peroxidase procedure by using the Ultravision Polyvalent (Rabbit-Mouse) Horseradish Peroxidase (HRP) Kit, 125 ml from Thermo Fisher Scientific (Waltham, MA, USA). The sections were incubated with TRAP (rabbit anti-TRAP/TRAP polyclonal antibody from Abcam (Cambridge, MA, USA)) and ALP (anti-ALP antibody, 100 ml from Abcam (Cambridge, MA, USA)) primary antibodies, overnight at 4 °C. After rinsing thoroughly with PBS, the sections were incubated with a biotinylated secondary antibody; after that, the horseradish peroxidase-conjugated streptavidin solution was added and incubated at room temperature for 10–15 min. Finally, the sections were visualized with diaminobenzidine substrate (DAB) (diaminobenzidine chromogen and substrate system, 125 ml from Thermo Fisher Scientific (Waltham, MA, USA)) as chromogen for 3–5 min at room temperature. Counterstaining was performed using hematoxylin, and the slides were visualized under a light microscope. The slides were processed immunohistochemically at the same laboratory conditions in order to obtain comparable staining intensities.

### Quantitative immunohistochemistry

**Fig. 4:**
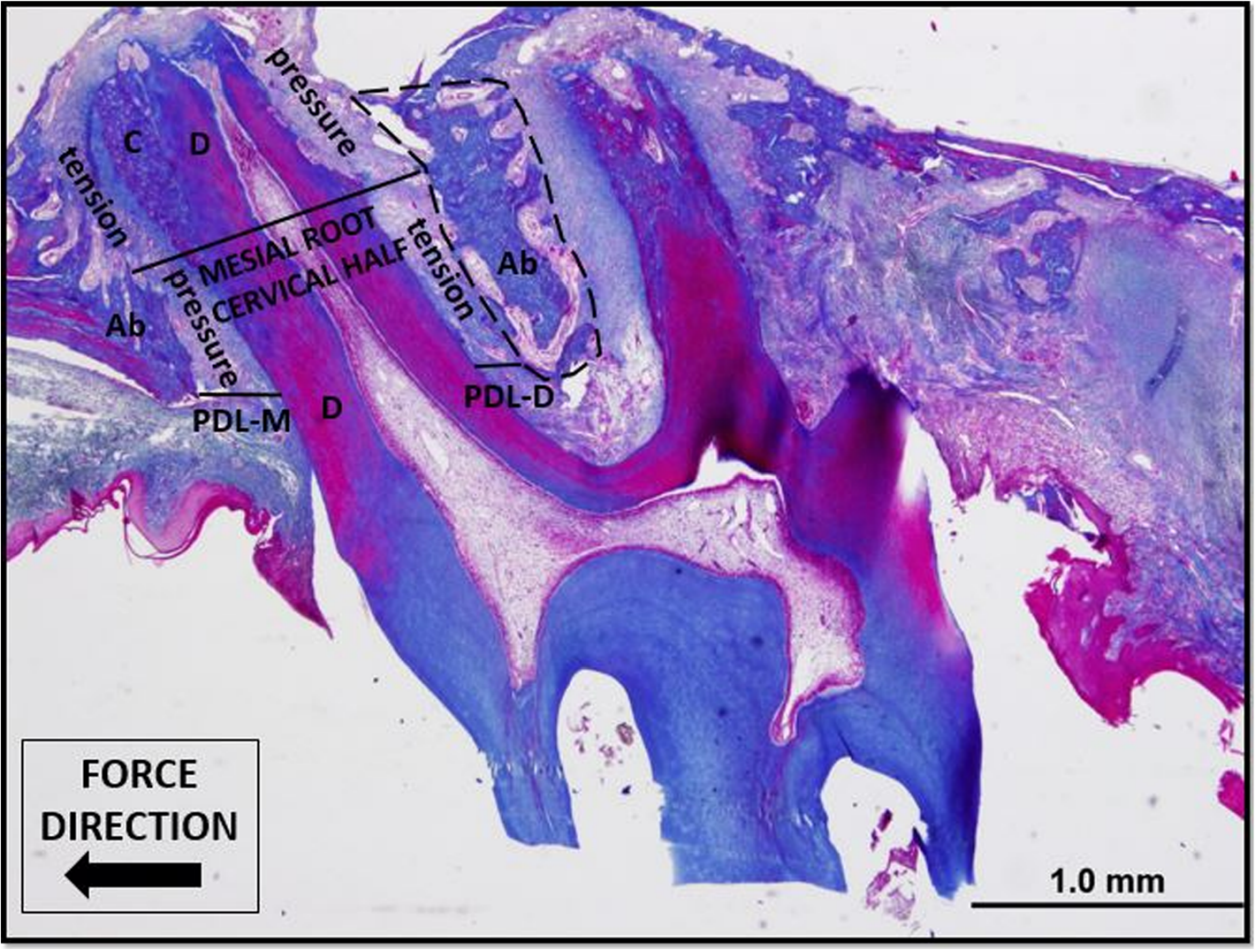
Demonstration of the PDL distance measurements at × 4 magnification by Masson trichrome staining. The pressure and tension regions of the cervical half of the mesial root where TRAP and ALP immunoreactivity density measurements were made are also shown. The area where the bone area measurement is performed is shown by dashed lines. Ab, alveolar bone; D, dentin; C, cementum

The digital color images were registered using a light microscope (Olympus BX-51, Japan) equipped with a camera (DP 71) and connected to a computer at × 10 and × 20 magnification from the mesial root region of the maxillary 1st molar tooth. Then, the quantification of the TRAP and ALP immunoreactivity intensity was calculated in both the mesial and the distal PDL regions where the middle of the root up to the cervical bone, and the results were recorded. Because we think that the laser we applied may not reach the apical region, we measured only the cervical half of the root. In our study, the cervical semi-pressure region in the mesial root in the mesial and the cervical half in the distal of the mesial root was used as the tension zone (Fig. 4).

### Statistical analysis

All of the data were transferred to SPSS 24.0 (SPSS Inc., Chicago, IL, USA) and prepared for the analysis. The normality of the variables was determined by using the Shapiro-Wilk test and homogeneities by using the Levene test. The data of PDL length, orthodontic tooth movement, and the bone area between the roots were evaluated using the non-parametric test, and the Kruskal-Wallis test was used for comparisons between the groups. The Mann-Whitney *U* test was used for the parameters which were statistically significant. *p* < 0.05 was considered statistically significant. The one-way ANOVA test was used for the analysis of TRAP and ALP immunoreactivity intensity data, and the Tukey HSD test was used as a post hoc analysis.

## Results

All animals survived to the end of the study. Weight loss was statistically significant in all animals 24 h after the dental movement apparatus was applied. In the following days, the animals gained weight regularly.

### Amount of the molar mesialization

Experimental tooth movement was obtained successfully in all groups; there was a statistically significant difference when compared with the negative control group (*p* < 0.001). The amount of molar mesialization was significantly higher in the 650 nm LLLT group than in the positive control group (*p* = 0.017) and 405 nm LLLT group (*p* = 0.042). There was no statistically significant difference between the other groups (Table 1).

**Table 1:** Evaluation of the amount of molar mesialization between the groups (mm)

**Group/variable**	**Molar mesialization (mm)**						**95% confidence interval for mean**							*p*	
	**Mean**			**S.D.**			**Lower bound**				**Upper bound**				
**NC**	0.000			0.000			0.000				0.000			< 0.001*	
**PC**	0.467			0.357			0.192				0.741				
**405 nm**	0.644			0.261			0.443				0.845				
**532 nm**	0.731			0.173			0.598				0.864				
**650 mm**	0.878			0.201			0.724				1.032				
**940 nm**	0.723			0.230			0.559				0.887				
**Pairwise comparisons,** ***p*** **values**															
**MM**	**NC-PC**	**NC-405 nm**	**NC-532 nm**	**NC-650 nm**	**NC-940 nm**	**PC-405 nm**	**PC-532 nm**	**PC-650 nm**	**PC-940 nm**	**405–532 nm**	**405–650 nm**	**405–940 nm**	**532–650 nm**	**532–940 nm**	**650–940 nm**
	0.000*	0.000*	0.000*	0.000*	0.000*	0.331	0.070	0.017*	0.165	0.251	0.042*	0.462	0.270	0.513	0.683

*MM* amount of molar mesialization, *NC* negative control group, *PC* positive control group, *S.D.* standard deviation

Significant degree: *p < 0.05

### PDL distances

There was a statistically significant difference in PDL-M and PDL-D values between the groups (*p* < 0.05). PDL-M distance was significantly shorter in the negative control group than in all other groups (*p* = 0.042) except for the 532 nm LLLT group (*p* = 0.097) (Table 2).

**Table 2:** Measurement findings of PDL distance (μm)

Group/variable		Distance of PDL		95% confidence interval for mean		*p*		Group/variable		Distance of PDL		95% confidence interval for mean			*p*
		Mean	S.D.	Lower bound	Upper bound					Mean	S.D.	Lower bound		Upper bound	
**PDL-M**	**NC**	0.178	0.009	0.169	0.188	0.042*		**PDL-D**	**NC**	0.136	0.015	0.120		0.152	0.007*
	**PC**	0.266	0.066	0.211	0.320				**PC**	0.225	0.048	0.185		0.266	
	**405 nm**	0.257	0.084	0.187	0.328				**405 nm**	0.198	0.060	0.148		0.248	
	**532 nm**	0.228	0.075	0.135	0.322				**532 nm**	0.179	0.018	0.157		0.202	
	**650 nm**	0.244	0.051	0.197	0.291				**650 nm**	0.218	0.063	0.161		0.276	
	**940 nm**	0.252	0.025	0.229	0.275				**940 nm**	0.153	0.029	0.127		0.180	
**Pairwise comparisons,** ***p*** **values**															
**Group/variable**	**NC-PC**	**NC-405 nm**	**NC-532 nm**	**NC-650 nm**	**NC-940 nm**	**PC-405 nm**	**PC-532 nm**	**PC-650 nm**	**PC-940 nm**	**405–532 nm**	**405–650 nm**	**405–940 nm**	**532–650 nm**	**532–940 nm**	**650–940 nm**
**PDL-M**	0.002*	0.027*	0.097	0.015*	0.003*	0.599	0.462	0.728	1.000	0.769	0.817	0.816	0.684	0.371	0.798
**PDL-D**	0.010*	0.038*	0.017*	0.031*	0.315	0.206	0.028*	0.954	0.015*	0.607	0.416	0.082	0.062	0.088	0.035*

*PDL-M* PDL distance in mesial-cervical, *PDL-D* PDL distance in distal-cervical, *NC* negative control group, *PC* positive control group, *S.D.* standard deviation

Significant degree: *p < 0.05

PDL-D distance was significantly shorter in the negative control group than in all other groups except for the 940 nm LLLT group (*p* = 0.007). PDL-D distance was significantly longer in the 532 nm (*p* = 0.028) and 940 nm (*p* = 0.015) LLLT groups than in the positive control group. In the 650 nm LLLT group, it was also significantly longer than in the 940 nm LLLT group (*p* = 0.035) (Table 2).

### Bone area between the roots

In terms of the bone area between the roots, there was a statistically significant difference between the groups (*p* < 0.05). The bone area in the 650 nm LLLT group was significantly higher than that in the NC (*p* = 0.022) and 405 nm LLLT (*p* = 0.036) groups. The bone area in the groups 650 nm LLLT and 940 nm LLLT was significantly higher than that in the negative control (*p* = 0.003) and 405 nm LLLT (*p* = 0.015) groups (Table 3).

**Table 3:** Findings of the bone area measurement between the roots (μm^2^)

**Group/variable**	**The bone area (BA)**						**95% confidence interval for mean**							*p*	
	**Mean**			**S.D.**			**Lower bound**				**Upper bound**				
**NC**	0.351			0.059			0.289				0.413			0.020*	
**PC**	0.467			0.135			0.354				0.581				
**405 nm**	0.407			0.090			0.332				0.483				
**532 nm**	0.447			0.106			0.316				0.579				
**650 mm**	0.502			0.098			0.411				0.593				
**940 nm**	0.594			0.124			0.480				0.709				
**Pairwise comparisons,** ***p*** **values**															
**BA**	**NC-PC**	**NC-405 nm**	**NC-532 nm**	**NC-650 nm**	**NC-940 nm**	**PC-405 nm**	**PC-532 nm**	**PC-650 nm**	**PC-940 nm**	**405–532 nm**	**405–650 nm**	**405–940 nm**	**532–650 nm**	**532–940 nm**	**650–940 nm**
	0.244	0.604	0.066	0.022*	0.003*	0.247	0.557	0.486	0.104	0.768	0.036*	0.015*	0.368	0.120	0.108

*BA* bone area between the roots, *NC* negative control group, *PC* positive control group, *S.D.* standard deviation

Significant degree: *p < 0.05

### TRAP staining immunoreactivity intensity

**Fig. 5:**
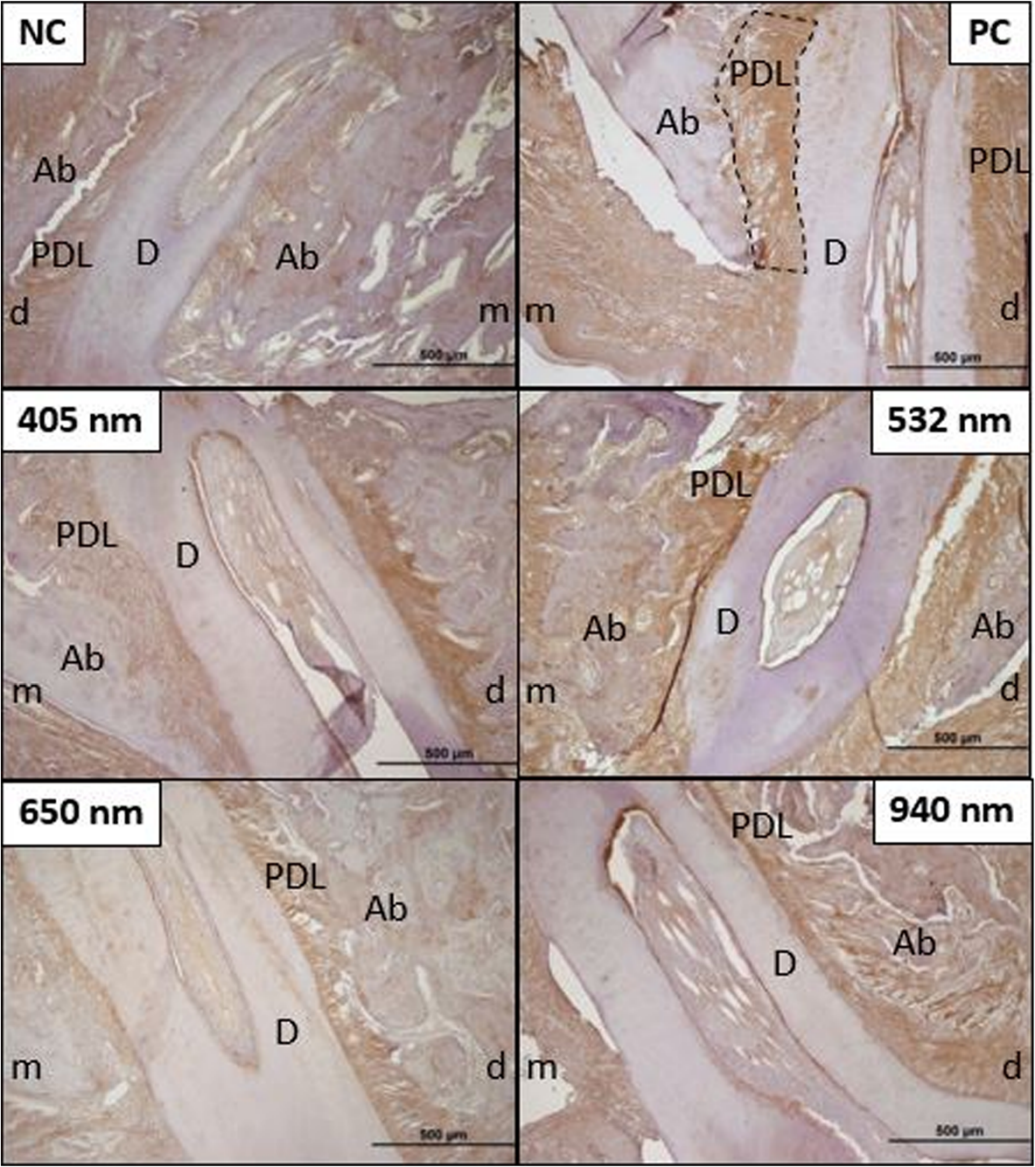
Preparations using the immunohistochemistry staining method with TRAP primary antibody for immunoreactivity intensity are shown in × 10 magnification. The black dashed line is marked to show the area of PDL in which the immunoreactivity measurement is performed (NC, negative control group; PC, positive control group; B, 405 nm LLLT group; C, 532 nm LLLT group; D, 650 nm LLLT group; E, 940 nm LLLT group; m, mesial; d, distal; PDL, periodontal ligament; Ab, alveolar bone; D, dentin)

Histological sections of TRAP staining for each group are shown in Fig. 5. In the measurements of the cervical half of the mesial root after TRAP staining, the mean immunoreactivity intensity in the mesial (TRAP-M) showed a significant difference between the groups (*p* = 0.002). There was no statistically significant difference in the distal side (TRAP-D). The immunoreactivity intensity for TRAP-M was significantly higher in the positive control group than in the 405 nm LLLT (*p* = 0.012) and 650 nm LLLT (*p* = 0.001) groups. No significant difference was found between the other groups (Table 4).

**Table 4:** Findings of immunoreactivity density of TRAP staining of the cervical half of the mesial root

**Group/variable**		**Immunoreactivity density**		**95% confidence interval for mean**			*p*	**Group/variable**		**Immunoreactivity density**		**95% confidence interval for mean**			*p*
		**Mean**	**S.D.**	**Lower bound**	**Upper bound**					**Mean**	**S.D.**	**Lower bound**		**Upper bound**	
**TRAP-M**	**NC**	97,489.83	10,017.30	86,977.32	108,002.34		0.002*	**TRAP-D**	**NC**	99,006.67	9034.43	89,525.62		108,487.72	0.188
	**PC**	109,420.33	8769.17	100,217.65	118,623.02				**PC**	113,892.17	18,793.44	94,169.67		133,614.67	
	**405 nm**	91,678.83	7313.39	84,003.90	99,353.77				**405 nm**	98,290.83	13,332.86	84,298.86		112,282.81	
	**532 nm**	94,642.00	7232.19	87,052.28	102,231.72				**532 nm**	96,798.67	12,373.72	83,813.24		109,784.09	
	**650 mm**	87,169.17	4934.65	81,990.56	92,347.77				**650 mm**	96,944.50	11,276.09	85,110.97		108,778.03	
	**940 nm**	98,521.00	11,046.92	86,927.97	110,114.03				**940 nm**	10,2087.83	7718.58	93,987.68		110,187.99	
**Pairwise comparisons,** ***p*** **values**															
**TRAP-M**	**NC-PC**	**NC-405 nm**	**NC-532 nm**	**NC-650 nm**	**NC-940 nm**	**PC-405 nm**	**PC-532 nm**	**PC-650 nm**	**PC-940 nm**	**405–532 nm**	**405–650 nm**	**405–940 nm**	**532–650 nm**	**532–940 nm**	**650–940 nm**
	0.174	0.838	0.991	0.308	1.000	0.012*	0.052	0.001*	0.254	0.990	0.937	0.726	0.648	0.966	0.216

*TRAP-M* immunoreactivity density of TRAP staining in the mesial side, *TRAP-D* immunoreactivity density of TRAP staining in the distal side, *NC* negative control group, *PC* positive control group, *S.D.* standard deviation

Significant degree: *p < 0.05

### ALP staining immunoreactivity intensity

**Fig. 6:**
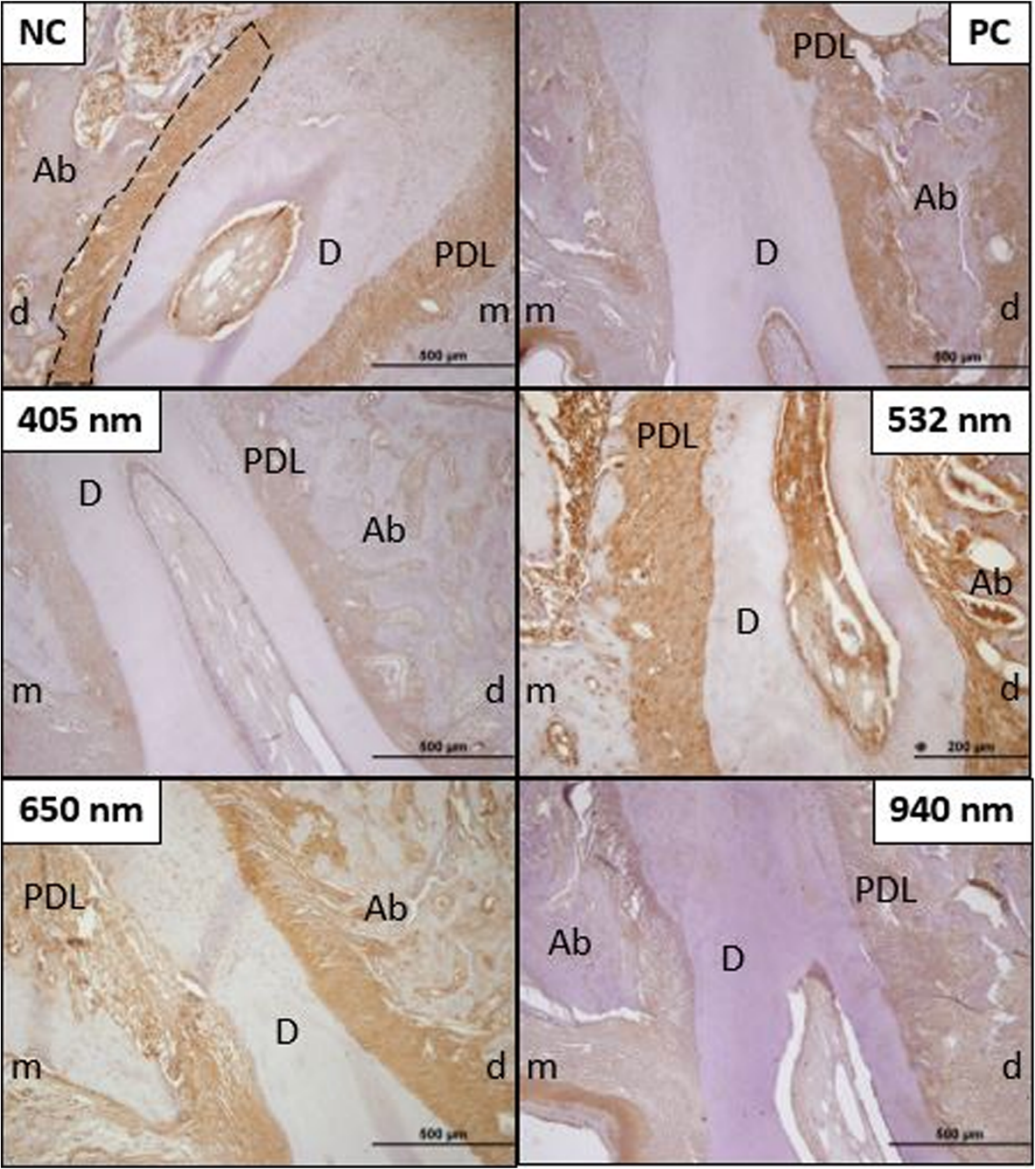
Preparations using the immunohistochemistry staining method with ALP primary antibody for immunoreactivity intensity are shown in × 10 magnification. The black dashed line is marked to show the area of PDL in which the immunoreactivity measurement is performed (NC, negative control group; PC, positive control group; B, 405 nm LLLT group; C, 532 nm LLLT group; D, 650 nm LLLT group; E, 940 nm LLLT group; m, mesial; d, distal; PDL, periodontal ligament; Ab, alveolar bone; D, dentin)

Histological sections of ALP staining according to groups are shown in Fig. 6. There was no statistically significant difference between the groups in terms of mean immunoreactivity intensity in both mesial and distal measurements in the cervical half of the mesial root after ALP staining (*p* < 0.05) (Table 5).

**Table 5:** Findings of immunoreactivity density of ALP staining of the cervical half of the mesial root

Group/variable		Immunoreactivity density		95% confidence interval for mean		*p*	Group/variable		Immunoreactivity density		95% confidence interval for mean		*p*
		Mean	S.D.	Lower bound	Upper bound				Mean	S.D.	Lower bound	Upper bound	
**ALP-M**	**NC**	91,407.33	9956.05	80,959.10	101,855.56	0.061	**ALP-D**	**NC**	94,242.00	11,511.02	82,161.93	106,322.07	0.684
	**PC**	99,344.50	18,454.73	79,977.44	118,711.56			**PC**	95,137.17	18,492.85	75,730.11	114,544.22	
	**405 nm**	92,642.83	13,129.49	78,864.28	106,421.39			**405 nm**	99,018.83	22,370.35	75,542.59	122,495.08	
	**532 nm**	111,564.00	11,419.06	99,580.43	123,547.57			**532 nm**	108,767.00	2929.26	105,692.93	111,841.07	
	**650 mm**	100,152.33	14,803.11	84,617.42	115,687.25			**650 mm**	101,704.83	21,937.68	78,682.65	124,727.01	
	**940 nm**	87,006.00	13,262.68	73,087.67	100,924.33			**940 nm**	95,051.17	18,401.12	75,740.28	114,361.95	

*ALP-M* immunoreactivity density of ALP staining in the mesial side, *ALP-D* immunoreactivity density of ALP staining in the distal side, *NC* negative control group, *PC* positive control group, *S.D.* standard deviation

Significant degree: *p < 0.05

## Discussion

Our study is the first study to investigate and compare the effect of LLLT of four different wavelengths such as 405 nm, 532 nm, 650 nm, and 940 nm on orthodontic tooth movement at the same study. A significant tooth movement was obtained in all groups undergoing orthodontic force, and the amount of molar mesialization was higher in all groups treated with laser than in the non-laser group. There are several studies in the literature on the effect of LLLT on tooth movement. But, there is no any study investigating the effects of different wavelengths by using the same parameters on tooth movement. The lasers we use in our study had the same parameters other than the wavelengths.

According to the results of a study conducted on rabbits comparing 650-nm and 830-nm wavelengths, laser applications slowed down tooth movement [14]. The visible spectral range has bands equivalent to three primary colors: blue (380–440 nm), green (440–600 nm), and red (600–750 nm); near-infrared range (750–1100 nm) and short-wave infrared range (1550–2400 nm) [19]. Although the penetration depths of lasers of these wavelengths on the skin are similar, lasers with colors near-infrared can reach a little deeper [20]. Still, it is not easy to estimate the depth of penetration on structures such as the gums, alveolar bone, and periodontal ligament in the mouth.

There are many studies in the literature showing that between 780- and 830-nm wavelengths of the GaAlAs diode laser, which is near-infrared, accelerate orthodontic tooth movement [8, 13, 21]. The number of studies performed with diode lasers with 650-nm and 940-nm wavelengths that we used in our study is less, and there are no other studies comparing them with each other under the same conditions. Therefore, we preferred diode lasers with wavelengths of 650 nm (red) and 940 nm (near-infrared), which have been less studied in the literature. Our results showed that 650-nm LLLT significantly increased the amount of molar mesialization compared to the groups treated with only orthodontic tooth movement and 405-nm LLLT. Since the laser at 940-nm wavelength is in the same group with the near-infrared lasers used in the other studies, it is thought to have a similar effect with them. In addition, Esnouf et al. reported that the 850-nm laser with 100-mW output power lost 66% of its power after 1 mm penetration [22]. Therefore, it is estimated that 650-nm LLLT may have similar effects on orthodontic tooth movement with GaAlAs diode lasers with wavelengths of approximately 800 nm used in some other studies or may be relatively more effective due to less energy loss during tissue penetration.

PDL distance varies according to the remodeling stage. As a result of the orthodontic force applied, the PDL distance on the pressure side decreases first, later increases when the alveolar bone resorption begins. Brudvick and Rygh [23] reported that the PDL distance on the side of the pressure started to return to normal size on the 7th day after the orthodontic force was applied. In our study, similar to the literature, the PDL distance on the pressure side (PDL-M) of all groups with tooth movement was significantly higher than that of the negative control group. There was no significant difference between the negative control group and the 532 nm laser group. In the PDL-D region, PDL distance in all groups except the 940 nm LLLT group was significantly higher than that in the negative control group. The PDL distance in groups 532 nm LLLT and 940 nm LLLT is also shorter than the PC group. Wang et al. [24] stated that the 540 nm LED light increases RUNX-2 expression, and 420 nm, 660 nm, and 810 nm wavelength groups are not significantly different from each other. RUNX-2 functions to direct multipotent mesenchymal stem cells to skeletal chondrocyte/osteoblast cells. In addition, RUNX-2 also plays a role in limiting the terminal differentiation of osteoblasts to osteocytes, thus maintaining the number of active osteoblasts [25]. Considering the results of Wang et al., 532-nm LLLT may have increased osteoblastic activity more than LLLTs of other wavelengths. Similarly, 940-nm LLLT was thought to increase osteoblastic activity, although not as much as 532-nm LLLT. The PDL distance in the 940 nm LLLT group was significantly shorter than that in the 650 nm LLLT group. The amount of molar mesialization in the 650 nm LLLT group was greater than that in the 940 nm LLLT group (and all other groups). Therefore, both the tension and the distance in the PDL-D region are probably higher than the other laser groups. Even if the healing potentials (osteogenesis) of all LLLTs are the same, it is thought that it may take more time for the distance to return to its normal state in the 650 nm LLLT group, since the largest distance is in this group. PDL distance measurements in our study were performed on the 14th day, when the PDL was partially normalized.

Kawasaki and Shimizu reported that the 830 nm LLLT group applied during the experimental tooth movement reported that the newly formed bone area on the tension side is more than the control group [21]. Merli et al. investigated the effects of 670-nm LLLT on wound healing of the mouse femoral bone. At the end of the 14-day trial period, they reported that the newly formed bone in the laser group had more space and intensity than that in the control group [26]. Kushibiki et al. [27] investigated the effects of lasers of 405 nm (blue), 664 nm (red), and 808 nm (infrared) on 10 different cell types. They reported that intracellular reactive oxygen radicals increased in 405-nm light cells, and there was no difference in other groups. In another study conducted in vitro with 635-nm LED light, it was found that LED application reduced osteoclastogenesis by lowering intracellular reactive oxygen radicals. Therefore, it has been reported that there may be a conservative alternative treatment approach in osteoporosis treatment [28]. The bone area observed in our study was significantly higher in the 650 nm and 940 nm LLLT groups than in the negative control and 405 nm LLLT groups. Our findings are consistent with the previous literature.

The occurrence of orthodontic tooth movement depends on the release of osteoclasts and precursor cells, osteoclast differentiation, and the intensity of osteoclast activity on the surface of bone-PDL, depending on the individual’s resorption potential. Fujita et al. [8] studied the effects of 810-nm laser and 830-nm LED (light-emitting diode) on tooth movement. The pressure side in the laser group reported that the intensity of TRAP immunoreactivity in PDL was higher on the 2nd, 3rd, 4th, and 7th days than in the LED and control group. In our study, we did not see the stage of intense osteoclastic cell forming in the initial tooth movement because we sacrificed the subjects on day 14. There was no statistically significant difference on the distal side in terms of TRAP immunoreactivity intensity. Since we do not expect osteoclastic activity in the tension region, this finding is consistent with the literature. The intensity of immunoreactivity for TRAP in the mesial side is less in all laser groups than in the PC group and closer to the values in the NC group; in other words, osteoclastic activity on day 14 in laser groups is less than in the PC group. But only the difference between the 405 nm and 650 nm LLLT groups and the PC group is significant. It is thought that osteoclastic activity was stimulated at an earlier stage in the LLLT groups than in the PC group, and similarly, bone turnover may have started earlier than in the PC group.

The verification of the production of ALP indicates that the osteoblast differentiation has started [29]. Kushibiki and Awazu [30] investigated the effects of 405-nm light on osteogenesis. According to the results of the study, ALP activity, calcium, and calcium phosphate accumulation increased 5 days after laser application.

Kim et al. [31] reported that exposure of 647-nm red light on mouse mesenchymal stem cells which were cultured in the presence of osteogenic differentiation medium for 3 days promotes osteoblastic differentiation significantly. Jawad et al. [32], according to the results of their in vitro study with the laser at 940 nm wavelength, stated there was an increased ALP activity in the laser applied group compared to the control group. They reported that LLLT at 940 nm wavelength can contribute to the bone formation by stimulating osteoblast cells.

In our study, there was no statistically significant difference between the groups in terms of ALP immunoreactivity intensity in both mesial and distal. In studies in the literature, since the evaluations are generally made between the 1st and 7th days, ALP activity is increased, whereas in our study, it is thought that there is no difference because the evaluations are done on the 14th day.

### Limitations

It is known that tooth movement consists of several phases. Lee reported that 6 h after the application of force, the width of the PDL decreased on the pressure side, deformation was seen in the fibroblasts, the number of Howship’s lacunae increased, and the hyalinization tissue disappeared on the 3rd day [33]. Radunovic et al. Reported that the most active “remodeling” response was seen 7 days after the force was applied [34].

Since we wanted to investigate the effect of low-level lasers of different wavelengths on the amount of orthodontic tooth movement in our study, the subjects were sacrificed only at the end of the 14th day. Therefore, the histological and immunohistochemical data obtained show the results at the stage when periodontal tissues partially normalize. The data to be obtained as a result of the sacrifices to be performed on the 3rd and 7th days of the orthodontic force application might provide more detailed information especially about PDL distance and bone turnover.

## Conclusions

The results of this study show that LLLT with 650-nm wavelength increases orthodontic tooth movement more than 405-nm, 532-nm, and 940-nm LLLTs. The 940-nm and 650-nm LLLT also increases the bone area between the roots by more than 405-nm and 532-nm wavelengths.

## Data Availability

All data generated or analyzed during this study are included in this published article.

## Abbreviations

LLLTs: Low-level laser therapies
PDL: Periodontal ligament
TRAP: Tartrate-resistant acid phosphatase
ALP: Alkaline phosphatase
CI: Confidence interval
ATP: Adenosine triphosphate
NO: Nitric oxide
ROS: Reactive oxygen species
IL1-β: Interleukin 1 beta
IL-6: Interleukin 6
TNF-α: Tumor necrosis factor alpha
RANKL: Receptor activator of nuclear factor kappa-B ligand
RANK: Receptor activator of nuclear factor kappa-B
LED: Light-emitting diode
RUNX-2: Runt-related protein 2
